# Thoughts on Public Perception of Ideal Breast Shape

**DOI:** 10.1093/asjof/ojac042

**Published:** 2022-05-07

**Authors:** Denis Souto Valente, Rafaela Koehler Zanella

**Affiliations:** Federal University of Medical Sciences of Porto Alegre, Brazil; PUCRS, Porto Alegre, Brazil

We read with interest the article by Kelly et al in which the authors investigated the public perception of ideal breast shape using a crowdsourcing survey of 960 answers (60% male).^1^ The authors used this database to “help improve patient outcomes by pairing the patients with the best possible cosmetic outcome for them.” ^1^ We appreciate the authors’ contribution to the growing literature on breast surgery outcomes and commend their efforts to standardize crowdsourcing as an option for learning ideal overall preferences for specific anatomy. However, an essential question of their findings was not dealt in-depth in their manuscript:

Will a woman seeking breast surgery have the same aesthetic preferences as a 60% male population? In our humble opinion, the answer to this question will be no. The gender of the participants significantly influenced their esthetic judgment.^2^ As suggested by Brustkern et al, “this difference might be attributable to an evolutionary, biologically sex-specific decision regarding parental investment and reproduction behavior.” ^3^

Generalizability, also known as external validity, is the extent to which the findings of a study can be applied to other settings.^4^ It seems that a 60% male perception of ideal breast shape cannot be applied to the usual female patient searching for aesthetic breast surgery.
